# Turning the Curve Into Straight: Phenogenetics of the Spine Morphology and Coordinate Maintenance in the Zebrafish

**DOI:** 10.3389/fcell.2021.801652

**Published:** 2022-01-26

**Authors:** Carlos Muñoz-Montecinos, Adrián Romero, Vania Sepúlveda, María Ángela Vira, Karen Fehrmann-Cartes, Sylvain Marcellini, Felipe Aguilera, Teresa Caprile, Ricardo Fuentes

**Affiliations:** ^1^ Departamento de Biología Celular, Facultad de Ciencias Biológicas, Universidad de Concepción, Concepción, Chile; ^2^ Grupo de Procesos en Biología del Desarrollo (GDeP), Facultad de Ciencias Biológicas, Universidad de Concepción, Concepción, Chile; ^3^ Núcleo de Investigaciones Aplicadas en Ciencias Veterinarias y Agronómicas, Universidad de las Américas, Concepción, Chile; ^4^ Departamento de Bioquímica y Biología Molecular, Facultad de Ciencias Biológicas, Universidad de Concepción, Concepción, Chile

**Keywords:** spine, scoliosis, zebrafish, cilia, cerebrospinal fluid, Reissner fiber, CSF-cNs, inflammation

## Abstract

The vertebral column, or spine, provides mechanical support and determines body axis posture and motion. The most common malformation altering spine morphology and function is adolescent idiopathic scoliosis (AIS), a three-dimensional spinal deformity that affects approximately 4% of the population worldwide. Due to AIS genetic heterogenicity and the lack of suitable animal models for its study, the etiology of this condition remains unclear, thus limiting treatment options. We here review current advances in zebrafish phenogenetics concerning AIS-like models and highlight the recently discovered biological processes leading to spine malformations. First, we focus on gene functions and phenotypes controlling critical aspects of postembryonic aspects that prime in spine architecture development and straightening. Second, we summarize how primary cilia assembly and biomechanical stimulus transduction, cerebrospinal fluid components and flow driven by motile cilia have been implicated in the pathogenesis of AIS-like phenotypes. Third, we highlight the inflammatory responses associated with scoliosis. We finally discuss recent innovations and methodologies for morphometrically characterize and analyze the zebrafish spine. Ongoing phenotyping projects are expected to identify novel and unprecedented postembryonic gene functions controlling spine morphology and mutant models of AIS. Importantly, imaging and gene editing technologies are allowing deep phenotyping studies in the zebrafish, opening new experimental paradigms in the morphometric and three-dimensional assessment of spinal malformations. In the future, fully elucidating the phenogenetic underpinnings of AIS etiology in zebrafish and humans will undoubtedly lead to innovative pharmacological treatments against spinal deformities.

## Introduction

The vertebral column (VC) or spine is the main feature that defines vertebrate animals. It extends from the head to the pelvis and consists in a straight segmented structure formed by a series of stacked bones or building blocks called vertebrae. These bones are separated and supported by intervertebral disks, muscles and ligaments. Morphologically, each vertebra is composed of a vertebral body or centrum, which wraps the notochord, and vertebral arches (Fleming et al., 2015). This structure provides support and flexibility along the body axis, allowing the symmetric distribution of the body weight and giving stability in both normal posture and corporal movement (Rockwell et al., 1938). Additionally, VC protects the spinal cord and vascular elements and supports the thoracic cage, providing a scaffold for lungs and heart protection (Rockwell et al., 1938).

Along the antero-posterior axis, the human spine is divided into four portions or curves: cervical, thoracic, lumbar, and sacral. The thoracic and sacral portions are formed during the early prenatal period as a continuous curve, while the cervical and lumbar curves appear in the fetal stage. Thus, completing their development after birth in response to head movements and to sitting and walking positions (Bagnall et al., 1977; Panattoni and Todros, 1988; Choufani et al., 2009). Abnormalities in the development of these curves lead to the three most common congenital VC deformities: kyphosis (excessive outward curve of the thoracic region), lordosis (abnormal lumbar curvature), and scoliosis [a three-dimensional (3D) torsion deformity of the spine and trunk] (Lonstein, 1999; Good et al., 2011).

Scoliosis is the most common deformity of the VC in humans and represents a major public health issue (Lonner et al., 2013). In human patients, this malformation is characterized by a lateral curvature greater than 10° (measured by the Cobb angle on X-ray image) and, in some cases, rotational defects (Negrini et al., 2012). This pathology develops before birth as a failure in vertebral formation or segmentation (congenital scoliosis), or later during postnatal growth (i.e., neuromuscular or idiopathic). Among the different types of scoliosis, the adolescent idiopathic scoliosis (AIS) is the most common, representing 80% of cases worldwide. This deformity arises in healthy individuals, with the absence of structural spine defects. The term “idiopathic” means that the etiology under scoliosis is unknown, and “adolescent” indicates that this condition emerges in children older than 10 years old (Hresko, 2013).

While developmental and morphological differences exist between spines from distinct vertebrate species, a common evolutionary origin has been proposed for this skeletal structure. Indeed, the straight morphology of the VC is conferred by conserved molecules and signaling pathways orchestrating the establishment and maintenance of the spine 3D axial plane (Brand-Saberi and Christ, 2000; Alejevski et al., 2021). However, our understanding of the identity and function of the molecules regulating spine morphology remains elusive because of the lack of tools to develop genetically tractable models to link genomic sequences with AIS.

In this review, we will discuss how the current emergence of zebrafish (*Danio rerio*) phenotyping data is providing a comprehensive collection of tools and information to understand spinal deformities in vertebrates, such as AIS. Mutant phenotypes displaying body axis curves are valuable genetic resources to identify molecular and cellular mechanisms that shape spine formation and patterning. Human genomic resources, coupled with phenogenetic information generated by gene function studies in zebrafish, will reveal novel genotype-phenotype correlations during spinal architecture morphogenesis and maintenance.

## 1 Human Adolescent Idiopathic Scoliosis: Genetics of a Mysterious Early and Defective Phenotype of the Spine

Scoliosis affects 0.47–5.2% of the population depending on the geographic location, ethnicity and sex, showing a marked bias to females, both in prevalence and severity. The risk indicators reveal a 2:1 prevalence ratio between girls and boys, and a 10-fold greater chance of developing curves that require surgical treatment (Karol et al., 1993; Konieczny et al., 2013). Severe spine curves in adolescence lead to health problems in adulthood, such as aesthetic issues, quality of life, disability, back pain, breathing function, among others (Negrini et al., 2006). Depending on the severity of the curve, scoliosis can be treated with observation, physical therapy, bracing, or invasive surgical corrections (Rose and Lenke, 2007). Because the underlying biological cause of AIS is poorly understood, effective treatment options are limited.

Genetic and environmental factors contribute to causing AIS. Also, the sexual dimorphism observed in this pathology suggest a model in which hereditary factors are involved. Despite nearly five decades of investigations on AIS supporting the idea that this condition has a genetic basis, surprisingly little is known about the genetic variants involved in its etiology. Thus, our ability to link genomic sequences with AIS has been hampered by the lack of tools to develop genetically tractable models. Such a resource would allow to identify scoliosis heritability; thus, contributing to the variability in limited treatment options.

**FIGURE 1:**
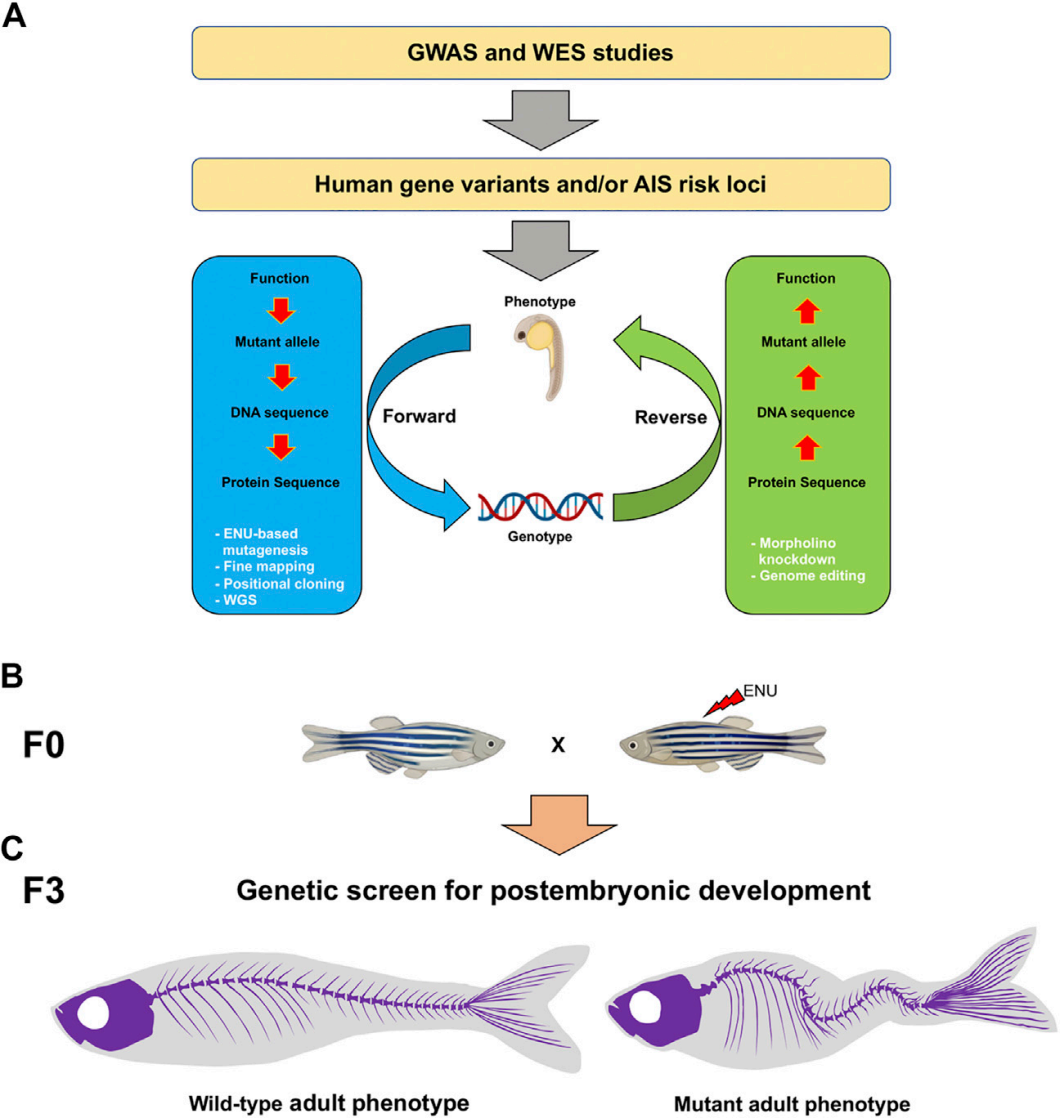
Schematic of the genotype-phenotype association and genetic approaches to study AIS candidate genes in the zebrafish. **(A)** To identify AIS risk loci, GWAS and WES strategies can be performed. Forward (blue) and reverse (green) genetic strategies (white) are powerful tools to study mutant genotypes and phenotypes associated with AIS. **(B)** In a forward genetic screen, male founders (F0) are mutagenized with ENU and then outcrossed to generate F2 families. To screen for postembryonic gene functions in late larval to adult stages after 5 dpf, F3 families are generated either through incrossing F2 heterozygous fish or through in vitro fertilization (IVF) using cryopreserved sperm and wild-type females, and raised to adulthood. **(C)** By using a combination of morphological, tomographic, molecular and cellular approaches, F3 individuals are screened for adult spine alterations. AIS, adolescent idiopathic scoliosis; GWAS, genome-wide association studies; WES, whole exome sequencing; ENU, N-ethyl-N-nitrosourea; WGS, whole genome sequencing.

In 1968, idiopathic scoliosis (IS) was described as a familial condition, because of its high incidence among relatives (Wynne-Davies, 1968). Studies accomplished on the Europeans twin population, revealed a higher concordance of AIS in monozygotes compared to dizygotes. This concordance for AIS in twins, strongly suggests a genetic contribution to the disease. Additionally, it has been shown that 38% of the variance in the risk to develop scoliosis is explained by additive genetic effects (Andersen et al., 2007; Grauers et al., 2012). In the recent years, several studies have focused on discovering the genetic variants associated with AIS, either by identifying mutations that segregate with the disease or polymorphic loci in affected population studies. Thus, genome-wide association studies (GWAS) and whole exome sequencing (WES) have greatly contributed to understanding the etiology of AIS by identifying single nucleotide polymorphisms (SNPs) and gene variants in several loci, respectively (Gao et al., 2007; Takahashi et al., 2011; Kou et al., 2013; Baschal et al., 2014; Ogura et al., 2015; Patten et al., 2015; Sharma et al., 2015; Zhu et al., 2015; Zhu et al., 2017; Khanshour et al., 2018; Kou et al., 2019; Liu et al., 2019; Wang et al., 2020; Terhune et al., 2021b). These efforts have been pivotal to characterize genotype-AIS associations and expand our understanding of the genetic regulation behind scoliosis in humans. However, these strategies are imprecise to identify the phenotypic consequences of genetic variants (Chanock et al., 2007; Linghu and DeLisi, 2010). Therefore, and considering that humans are unsuitable for robust functional interrogation of the largely unknown functions of AIS-associated genes at large scale, there is a need for model systems that enable the generation of mutants to study genotype-phenotype interactions. Indeed, the function of AIS risk genes identified by GWAS and WES studies, including LBX1, DNAAF1, ZMYND10, MAPK7, KIF6, and PAX1, has successfully been studied in a vertebrate mutant system (Figure 1A) (Adham et al., 2005; Guo et al., 2016; Gao et al., 2017; Konjikusic et al., 2018; Wang et al., 2020).

Although the idea that genetic factors contribute to scoliosis has strong supporting evidence, AIS inheritance does not behave as a simple Mendelian mechanism. It is thought that AIS is a polygenic condition with genetic heterogenicity, variable patterns of inheritance, and a mode of genetic transmission that still remains to be identified (Cheng et al., 2015). Even considering the last advances in genomic technologies applied to discover human AIS-related genes, these findings represent statistical association rather than causality. Currently, the available genetic tests are inaccurate enough to predict the prognosis of AIS; therefore, further investigation is needed to develop genetic tools for early detection, which would allow physicians to design better management plans for this condition (Zamborsky et al., 2019).

## 2 Zebrafish as a Model System to Understand Body Axis Linearity Maintenance and Human Adolescent Idiopathic Scoliosis

To study the causes of AIS in humans, diverse animal model systems have been used to date. Due to the low natural incidence of scoliosis in quadrupedal organisms, it has been proposed that upright position is critical in the development of spinal malformation (Machida et al., 1999; Castelein et al., 2005). Also, the mechanical load distribution along the VC differs between bipedal and quadrupedal animals, which impacts spine anatomy. However, how bipedal behaviors and adaptations are linked to scoliosis pathogenesis remains mysterious.

Because of the lack of non-human bipedal mammals and the low rate of spinal deformities in quadrupedal vertebrates, researchers have developed several procedures to induce bipedalism and scoliosis-like phenotypes in experimental animals. These include amputation of forelimbs and tail, or pinealectomy (reviewed in Ouellet and Odent, 2013). These approaches have also been applied to primates without any success (Robin and Stein, 1975). In our knowledge, many of these procedures aim to reproduce the curvatures of human scoliosis rather than its pathogenesis, thus contributing to testing corrective surgeries or mechanical treatments, instead of finding genetic causes underlying AIS in humans (Bobyn et al., 2015). As none of these experimental animals can recapitulate all the features of the pathogenic phenotype, it is paramount the emergence of additional model systems to model genotype-phenotype relationships across AIS development.

Recently, the molecular mechanisms underlying AIS have been better understood with the emergence of zebrafish as a suitable model for recapitulating human diseases. Using zebrafish and other teleost fish such as medaka and guppy, several research groups have achieved important advances in our understanding of AIS phenogenetics (Gorman et al., 2007; Hayes et al., 2014; Grimes et al., 2016; Kobayashi et al., 2017; Cantaut-Belarif et al., 2018; Van Gennip et al., 2018; Zhang et al., 2018; Rose et al., 2020). Unlike classical quadruped animal model systems; whose spinal loading and center of mass are different compared to humans (Gorman and Breden, 2009), zebrafish generates craniocaudal forces because of swimming, similar to those exerted by humans standing upright. These physicomechanical features, in addition to emerging genetic and phenotypic evidence from zebrafish, pose this animal model as suitable to study the role of molecular, cellular and physiological processes implicated in the etiopathogenesis of AIS (Buchan et al., 2014; Grimes et al., 2016; Van Gennip et al., 2018; Zhang et al., 2018; Rose et al., 2020). All these aspects will be discussed in the next sections.

## 3 Cellular Identity and Mechanisms of the Spine Axis Forming Activity in Zebrafish

In teleostean fishes, the building blocks of the spine arise from two embryonic structures: the notochord and somites. During early embryogenesis, somites are formed sequentially along the anterior-posterior (AP) axis from the paraxial mesoderm, which undergoes mesenchymal-to-epithelial transition, and is patterned by maternally defined signaling morphogen gradients such as Nodal, Wnt, and FGF (Dequeant and Pourquie, 2008; Tuazon and Mullins, 2015; Fuentes et al., 2020). Somites are sphere-like structures formed by epithelial cells, which localize on both sides of the neural tube in the developing embryo. Then, each somite subdivides into dermomyotome (precursors of the dermis and skeletal muscle) and the sclerotome (precursor of most ligaments, bones and cartilages of the spine) (Fleming et al., 2015). Alternatively, the notochord is also formed by AP patterning cues during early development, supporting the body axis and providing flexibility during locomotion until the spine is structured (Stemple, 2005). The notochord is formed by inner vacuolated cells called chondrocytes, and by an external layer of chondroblasts that secretes the notochordal sheath (a thin membrane with high elastin content enveloping a collagenous layer). This extracellular matrix is essential for the hydrostatic properties of the notochord, and forms a basal lamina on which chondroblasts rest (Grotmol et al., 2005).

The first step during zebrafish spine development is the formation of mineralized rings (called chordacentra) that surround the notochord sheath (Arratia et al., 2001; Fleming et al., 2015). The chordacentra mineralizes in the absence of osteoblasts, and serves as a scaffold for the developing autocentrum, a layer of perinotochordal bone formed at the periphery of the notochord by direct ossification (Arratia et al., 2001; Fleming et al., 2004; Peskin et al., 2020). During this process, the notochordal sheath is segmented, from a cartilage-like structure to alternated mineralized domains (Forero et al., 2018; Wopat et al., 2018). This transition occurs through the spatially restricted expression of *ectonucleoside triphosphate diphosphohydrolase 5* (*entpd5*) gene in the notochord sheath cells, and relies on the Notch pathway. Entpd5 functions to recruit osteoblasts to the mineralized zone and ultimately form the centra (Forero et al., 2018; Wopat et al., 2018). Depending on the position along the spine, the ventral and neural arches can develop from either cartilage or membrane bone. The most anterior (Weberian apparatus) and posterior (caudal fin) arches have a cartilaginous precursor, which is formed by metametric aggregations of sclerotomal mesenchyme (sclerotome). Central arches are formed by membranous ossification or by a combination of both (Bird and Mabee, 2003; Grotmol et al., 2003).

Although the spine is formed in all vertebrates from the sclerotome and notochord, there are important differences concerning the contribution of each embryonic tissue and the concomitantly spine formation between species. Some of these include the proportion of the somites, the segmentation pattern of the spine that relies on the segmentation clock patterning, and the segmental periodicity of vertebrae that is determined by the sclerotome instead of the notochord in some species. However, in teleost fish, the segmentation of the spine relies on the notochord, with contribution from the paraxial mesoderm (Morin-Kensicki and Eisen, 1997). Despite these differences, the general architecture of the spine is evolutionarily conserved among vertebrate groups and suggests the existence of multiscale and spatially defined signatures of its morphogenesis at the phenogenetic level (Boswell and Ciruna, 2017). Therefore, this evolutionary conservation opens the possibility to establish new model systems for investigating genotype-phenotype correlations at large-scale during spine formation. In this sense, the zebrafish emerges as an attractive model system for studying scoliosis and interrogating specific gene functions underpinning spine axis morphogenesis (Figure 1). Discovering novel genes involved in biological processes associated with the generation of scoliosis will also allow us not only to study this pathology in real-time, also to develop efficient genetic-based diagnostic or prognostic tools.

## 4 Emerging Roles of Genetic Factors Related to Scoliosis Development in Zebrafish

Phenotyping efforts in zebrafish based on forward and reverse genetic approaches (Figure 1A), along with recent advances in sequencing and genome-editing technologies, represent promising strategies to reveal the underlying causes of AIS in humans (Buchan et al., 2014; Grimes et al., 2016; Zhang et al., 2018; Rose et al., 2020). Thus, zebrafish mutants have revealed several genes related to scoliosis, most of these regulating three main and apparently linked biological processes: skeleton development, ciliogenesis [that impacts on cerebrospinal fluid (CSF) dynamics], and immune response.

### 4.1 Molecular Factors Involved in Spine Patterning and Maintenance

In the zebrafish early embryo, notochord formation is critical for the AP extension of the body axis, and ultimately, spine biogenesis. Signaling pathways, such as Notch, regulate notochord-lineage cell fate by determining if these differentiate into chondrocytes or chondroblasts (Yamamoto et al., 2010). As aforementioned, Notch signaling activation induces the transition from cartilage-like to mineralized domains by inducing the expression of Entpd5 as an essential factor for the notochord ossification (Huitema et al., 2012; Wopat et al., 2018). While the role of the Notch signaling in determining the onset of spine formation has been established, much less is known about the genetic program controlling how the spine develops from embryo to juvenile stages, and maintains its morphology and linearity in the adult individual.

The study of a collection of zebrafish mutants displaying scoliosis-like phenotypes has significantly increased our knowledge regarding the genetic regulation of spine development (Figures 1B,C). A forward genetic approach led to the identification of dozens of genes involved in axial skeleton morphogenesis during early embryogenesis (Brand et al., 1996; Sun et al., 2004; Henke et al., 2017; Gray et al., 2020). Regardless of the great contribution in the understanding of spine formation in the early embryo, postembryonic genetic analysis of this structure has received much less attention; thus constituting a gap in our knowledge of human pathologies, including AIS.

Pioneering large-scale screens in zebrafish have identified several adult mutant phenotypes (Driever et al., 1996; Haffter et al., 1996). Since then, phenotyping approaches have allowed the identification and characterization of a large number of genes functioning in axial skeleton development (Haffter et al., 1996). For instance, Stemple and colleagues reported the mutant known as *leviathan*, which shows a dorsoventral and lateral folded notochord (Stemple et al., 1996). Almost two decades later, three different recessive mutations in the *col8a1a* gene were associated with the *leviathan* genetic lesion (Gray et al., 2014). These mutant alleles also display vertebral malformations in the adult stage, becoming a suitable model for congenital scoliosis.

Unlike *leviathan* mutant, *skolios*, another zebrafish mutant with skeletal abnormalities, shares several features with human IS as it develops a curved body axis without vertebral deformations and bone homeostasis alterations. A nonsense mutation in the *kinesin family member 6* (*kif6*) gene, which functions in the cilia assembly, was identified in *skolios* mutants (Buchan et al., 2014). However, no typical ciliary dysfunctions such as hydrocephalus, kidney cyst or fluid flow at the central canal were observed in *kif6* mutants (Buchan et al., 2014), perhaps reflecting a highly tissue-specific function. Additionally, the molecular characterization of the zebrafish *protein tyrosine kinase 7* (*ptk7*) gene indicates a role of the Wnt/β-catenin and planar cell polarity signaling in patterning, establishing and maintaining axial coordinates during the juvenile-to-adult transition (Hayes et al., 2013; Grimes et al., 2016). *skolios*/*kif6* and *ptk7* were the first identified mutant genes to disrupt spine linearity; thus being pioneers in recapitulating AIS in zebrafish (Buchan et al., 2014; Grimes et al., 2016). Recently, it has been reported that a deficiency of *somite shorten* (*smt*) gene leads to failure in notochord function, which generates a scoliosis-like phenotype (Sun et al., 2020). The molecular identification of the zebrafish *smt* gene as *dual serine/threonine and tyrosine protein kinase* (*dstyk*), indicates a role of Smt/Dstyk in establishing spine axial coordinates through a lysosome-to-nucleus signaling mechanism (Sun et al., 2020). Altogether, these findings highlight the potential of zebrafish as a model system for understanding the onset of scoliosis, but there is much yet to be discovered at the genetic and molecular level.

### 4.2 Cilia Function and Spinal Curvature in Zebrafish

Cilia are evolutionarily conserved hair-like organelles, protruding from the cell membrane to the extracellular space. These cellular structures are present in unicellular and multicellular organisms, and can be found in many vertebrate cell types (Satir et al., 2008; Ishikawa and Marshall, 2011; Malicki and Johnson, 2017). At the structural level, cilia consist of a microtubule (MT) skeleton called axoneme that is surrounded by and docks to the plasma membrane through the basal body (Ishikawa and Marshall, 2011; Malicki and Johnson, 2017). Based on MT organization, cilia are classified either as motile or non-motile. The axoneme of motile cilia contains nine doublets surrounding a central pair of singlet MTs (9 + 2), and axonemal dynein arms that allow cilia movements catalyzed by ATP hydrolysis. Non-motile or primary cilia lack the central pair of singlet MTs (9 + 0) and do not contain dynein arms (Wang and Dynlacht, 2018). From a functional perspective, motile cilia generate forces for cell motility and can rhythmically beat to drive fluids to flow over epithelial cells, and/or generate signaling gradients. Primary, or non-motile, cilia act as sensors transducing chemical or physical signals from the pericellular environment into the cell (Wang and Dynlacht, 2018). Interestingly, cilia biology is critical for spine maintenance and impaired function is associated with scoliosis (reviewed in Boswell and Ciruna, 2017; Grimes, 2019; Bearce and Grimes, 2021).

The relevance of cilia in the maintenance of body axis straightness was initially described in the 1990s in large-scale N-ethyl-Nitrosourea (ENU)-based mutagenesis screens and more recently, by specifically mutating cilia-related genes (Driever et al., 1996; Haffter et al., 1996; Grimes, 2019). Thus, most of the curly tail down phenotypes have alterations in their cilia. Also, motile cilia have been described as critical for normal CSF flow (Kramer-Zucker et al., 2005; Grimes et al., 2016; Thouvenin et al., 2020), which is an important corporal fluid for vertebrate development (Lun et al., 2015), and required for early body axis straightening and its maintenance during zebrafish development (see Section 5.3) (Grimes et al., 2016; Cantaut-Belarif et al., 2018; Zhang et al., 2018; Troutwine et al., 2020).

One of the most remarkable associations between motile ciliopathy and scoliosis was conducted by Grimes et al. (2016). These authors analyzed the body curvature phenotype in five different cilia-related zebrafish mutants. They also generated two complementary strategies to avoid the early lethality caused by aberrant cilia motility in other mutants during the first weeks of development (Grimes et al., 2016). First, they used a temperature-sensitive mutation (*kurly*/*cfap298* mutant), allowing the embryo to develop normally when the temperature is shifted from a warmer to cooler conditions. These animals appear wild-type through the first development stages but develop spinal curves during late larval stages. The second strategy consisted of injecting wild-type RNA at 1-cell stage in animals from three different mutant backgrounds showing defective cilia motility. This rescue approach allowed early development to proceed normally, and revealed that older embryos developed curved spines (Grimes et al., 2016). One year later, Oliazadeh et al. (2017) studied the organization and morphology of primary cilia in bone cells from patients with AIS, describing an increase in the length with cellular consequences related to mechanotransduction, that in turn affected bone formation and development (Nguyen and Jacobs, 2013; Oliazadeh et al., 2017).

In addition to *ptk7* (see Section 4.1), several motile ciliary zebrafish genes loss-of-function resemble human ciliopathy phenotypes, including curved body axis (reviewed in Song et al., 2016). Alternatively, it is also well known that the sensory primary cilia function determines the coordinated activation of signaling pathways such as Hedgehog, WNT, TGF-β/BMP, among others (Anvarian et al., 2019). However, how mutations in cilia structure protein-coding genes may generate alterations in spine biology remains an unsolved problem. In addition, while these results put in evidence the direct relationship between cilia function perturbance and scoliosis, the involved cilia (primary cilia, motile cilia or both) remains controversial.

For example, Cep290 (centrosomal protein 290) promotes primary cilia assembly and localizes at centrioles and basal bodies. Mutations in this gene have been implicated in different human ciliopathies, including Joubert syndrome, which is characterized by hindbrain malformation, and motor and cognitive impairments (Saraiva and Baraitser, 1992). Zebrafish *cep290* morphants resemble human ciliopathies such as body axis curvature, defects in cerebellar development, cystic kidney and reduced vision (Sayer et al., 2006; Baye et al., 2011). Additionally, adult *cep290* mutants exhibit a severe spine malformation and are unable to breed (Lessieur et al., 2019), thus suggesting an important role of primary cilia in zebrafish reproduction.

Additionally, the human Armc9 (armadillo repeat containing 9) protein has recently been associated with primary cilia function and ciliopathies (Breslow et al., 2018). Armc9 localizes at the proximal region of cilia and regulates their length in mammalian cells. On the other hand, Van De Weghe et al. (2017) found that this protein is also mutated in individuals with Joubert syndrome. Remarkably, zebrafish *armc9 crispant* shows a decreased number of cilia in brain ventricles, and typical ciliopathy phenotypes, including scoliosis-like spinal curvature (Van De Weghe et al., 2017).

The role of cilia in spine coordinate establishment and morphology is increasing the spectrum of genotype-phenotype relationships in vertebrate post-embryonic development so far. The use of zebrafish seems advantageous in the study of primary and motile cilia structure and how their functions contribute to body axis development and the maintenance of its straightening. Ongoing phenotyping projects in zebrafish are expected to provide unprecedented genetic entry points to cilia biology and spine morphogenesis.

### 4.3 Mechanism of Cerebrospinal Fluid Flow Establishment: A Hydrodynamic Model Predicting the Spine Axial Coordinates and Curvature

CSF is a clear liquid filling the cerebral ventricles and central canal of the spinal cord. This fluid also surrounds the central nervous system (CNS) in the subarachnoid space (Brinker et al., 2014). CSF functions: a) providing buoyancy and protection to the brain and spinal cord, and b) transporting several signals along with the central nervous system (Brinker et al., 2014; Fame and Lehtinen, 2020). The CSF and its correct circulation are also crucial for normal CNS development, providing growth factors and signaling molecules that impact neuroepithelium proliferation and differentiation.

The “classic CSF circulation theory” presumes a constant downward flow from the lateral ventricles toward the subarachnoid space. This theory describes CSF flow as unidirectional, circulating from the lateral to the third ventricle via the foramen of Monro, and then through the Sylvian aqueduct to the fourth ventricle. Subsequently, the CSF flows caudally along the central canal of the spinal cord or circulates through the foramens of Luschka and Magendie into the subarachnoid space of the brain and spinal cord, where a portion of CSF drains to the blood via arachnoid granulation, via spinal nerve roots and the remainder via the olfactory tracts (Brodbelt and Stoodley, 2007).

Recently, the use of molecular, cell biology, and neuroimaging techniques has revealed that CSF flow is not as simple as previously thought. This flow depends on multiple factors including cilia motility, hearth beat, and pulsatile and local exchange between interstitial fluid, blood, and CSF (Brinker et al., 2014; Matsumae et al., 2019; Fame and Lehtinen, 2020). Although most of the current knowledge about CSF flow corresponds to its dynamics in the brain, much less is known about its behavior within the central canal of the spinal cord.

In vertebrates, the central canal of the spinal cord is a narrow cylindrical cavity surrounded by ependymal cells. It extends caudally from the floor of the fourth ventricle to the terminal ventricle or ampulla caudalis, at the conus medularis (Storer et al., 1998). The central canal is limited by ependyma ciliated cells whose asymmetric distribution generates a recently described bidirectional fluid flow (Thouvenin et al., 2020). At 30 h post-fertilization, zebrafish embryos display a density of ventral motile cilia, which is four times higher than the dorsal ones and beat with higher frequency (Troutwine et al., 2020). This asymmetry generates a dorsocaudal CSF flow at the ventral region of the central canal and the opposite direction at the dorsal region. In the absence of cilia motility, the central canal collapses, indicating that the ciliary activity is necessary to maintain its morphology and the transport of molecules between the brain and spinal cord (Troutwine et al., 2020).

**FIGURE 2:**
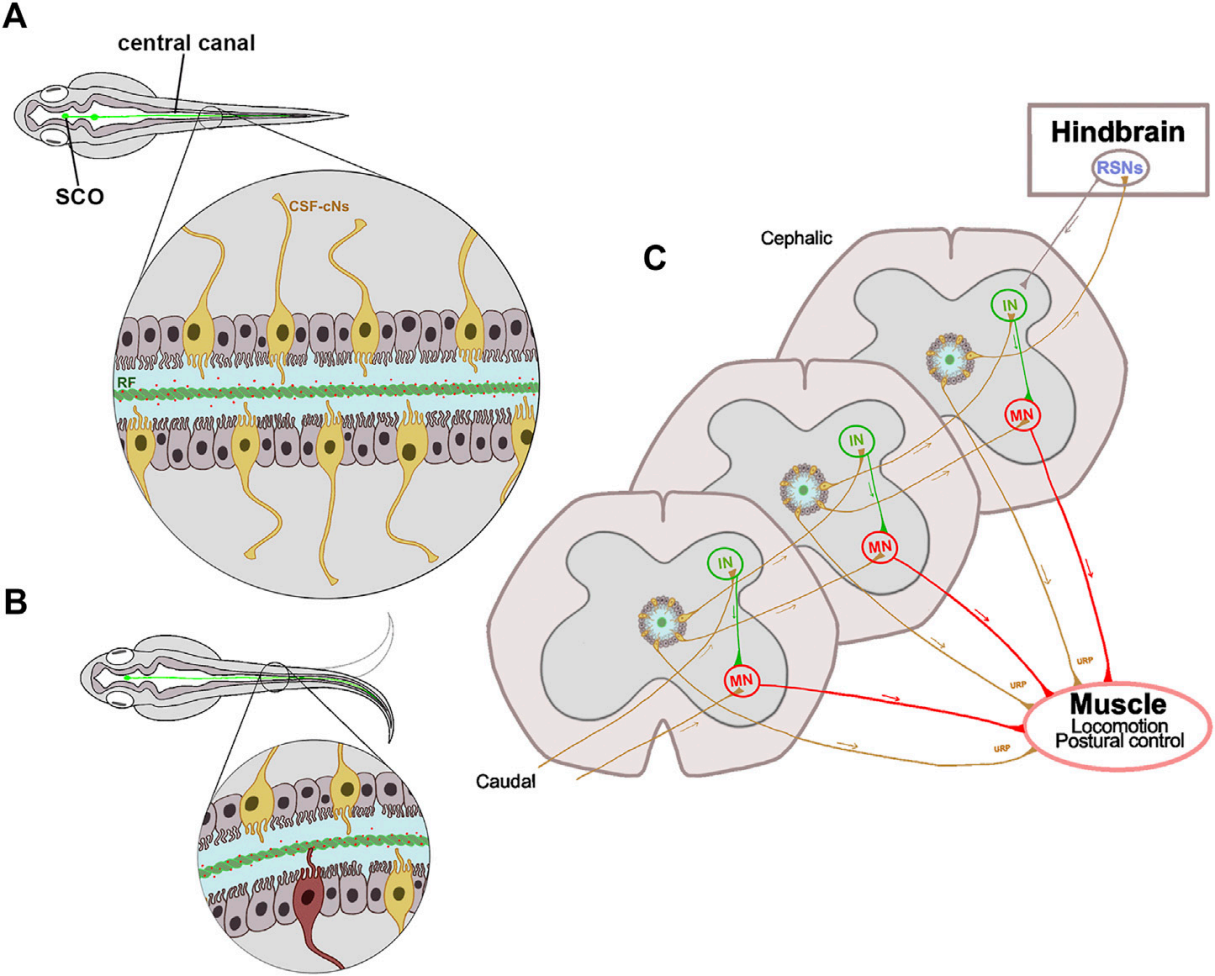
Schematic representation of the proprioceptive organ conformed by RF and CSF-cNs. **(A)** The central canal in a zebrafish embryo is surrounded by ependymal ciliated cells (gray) necessary for the CSF flow, and by sensory CSF-cNs (yellow). The RF (green cord) runs from the SCO to the entire spinal cord inside the central canal. The RF binds and transports epinephrine and norepinephrine molecules (red dots) that trigger Urp1 and Urp2 expression in mechanosensory CSF-cNs. **(B)** The curvature of the spinal cord during locomotion, allows the interaction between RF and CSF-cNs (highlighted in red); thus, transferring information through a spinal circuit and shaping the spinal curvature. **(C)** CSF-cNs project an ipsilateral ascending axon that synapses onto premotor INs, onto primary MNs or directly onto muscle that expresses the Urp receptor. Additionally, the most cephalic CSF-cNs send their axons to RSNs in the hindbrain, that in turn send descending motor commands to the spinal cord. All these projections participate in the correct muscle contraction and postural control. SCO, subcommissural organ; CSF, cerebrospinal fluid; CSF-cNs, CSF contacting neurons; RF, Reissner fiber; Urp, urotensin neuropeptides; IN, interneuron; MN, motor neuron; RSN, reticulospinal neuron.

In addition to the CSF, the central canal contains a threadlike structure, called Reissner fiber (RF) (Figure 2A). This fiber is formed from the early stages of development in most vertebrates, although its presence in man and bats is still controversial (Rodriguez et al., 1992). Over the last years, and using the zebrafish as an animal model system, several studies have identified genetic factors associated with body axis patterning and defective functions of the CSF flow, motile cilia or RF formation. These findings are starting to fill the gap in our knowledge about the link between CNS functionality and scoliosis progression, as reviewed below.

#### 4.3.1 Relevance of Cerebrospinal Fluid Flow, Reissner Fiber Biogenesis and Spine Morphogenesis in Zebrafish

In zebrafish, CSF circulation in the spinal cord is critical for spine morphogenesis, and disturbances in its dynamics have been associated with scoliosis (Grimes et al., 2016; Boswell and Ciruna, 2017; Zhang et al., 2018; Grimes, 2019; Thouvenin et al., 2020). The relevance of a normal CSF flow inside the central canal to keep a straight body axis is emerging from the zebrafish model, but how do CSF flow perturbances generate scoliosis? To address this question, there are at least two non-exclusive hypotheses that will be discussed here.

Firstly, the CSF provides signaling resources necessary for correct body axis maintenance. CSF flow is driven by motile cilia on ependymal cells of brain ventricles and central canal. As discussed in Section 4.2, these MT-based structures emerge from the apical cell surface and, by a synchronized beat, generate a wave-like movement of the extracellular fluid (Figure 2). In this way, the injection of fluorescent dyes in the brain ventricles of zebrafish embryos led to a rapidly dispersion of fluorescence along the spinal canal. However, dye transport is impaired in ciliary mutants, and correlates with the severity of body curvature (Zhang et al., 2018). The *ptk7* mutant shows abnormal cilia structure and organization, as well as CSF flow defects, hydrocephalus and scoliosis (Grimes et al., 2016). These defective phenotypes can be rescued when expressing the Ptk7 wild-type version in motile ciliated cell lineages. Thus, these findings indicate that motile cilia function is necessary to maintain a correct CSF flow and right body axis (Grimes et al., 2016).

To characterize the molecular pathways affected by the CSF flow perturbance, Zhang and colleagues elegantly compared gene-expression patterns between control and *zmynd10* mutant embryos (Zhang et al., 2018). The Zmynd10 protein functions assembling the arms of axonemal dynein and mutations on its coding gene cause ciliopathy primary ciliary dyskinesia (PCD) in humans (Zariwala et al., 2013). The results showed that two genes, urotensin-related peptide 1 and 2 (Urp1 and Urp2) were downregulated in the *zmynd10* mutant, as well as in other cilia mutants displaying a body curvature phenotype (Zhang et al., 2018). The relevance of these peptides was then analyzed by gain- and loss-of-function assays. In gain-of-function assays, these researchers achieved to rescue body axis curvature in different cilia mutants by overexpressing Urp1 (or Urp2). In loss-of-function assays, the generation of *urp1* morphant embryos develop a body-curvature defect, and the phenotype was more pronounced when *urp2* morpholine was co-injected. These results show that body curvature alteration found in motile cilia mutants is caused by downregulation of Urps. These peptides are secreted by CSF-contacting neurons (CSF-cNs) (Figure 2C), and activate the Uts2ra receptor in somitic muscle cells; thus, regulating body axis extension. In this way, norepinephrine can restore the expression of Urp1 and right body axis in motile cilia mutants but not in *urp1* or *utsra* receptor morphants (Zhang et al., 2018). These findings indicate that the transport of molecules by the CSF is relevant to maintain the straight body axis (Figures 2A,B).

Secondly, CSF flow is necessary for the correct aggregation of RF components. RF is an elastic extracellular structure suspended in the CSF and extended from the diencephalon to the entire length of the central canal of the spinal cord (Figure 2A) (Rodriguez et al., 1998; Troutwine et al., 2020). RF is evolutionarily conserved, and its main component is a protein secreted to the CSF from early stages of development by the subcommissural organ (SCO), a brain gland located at the diencephalic-mesencephalic boundary in all vertebrates (Rodriguez et al., 1992) (Figure 2A). This protein is known as the SCO-spondin, a glycoprotein with a high molecular mass (540.67 KDa), and broadly conserved across vertebrates (Meiniel and Meiniel, 2007; Munoz et al., 2019). Once released into the CSF, SCO-spondin undergoes a progressive process of aggregation. Primarily, as pre-RF by forming a mesh of fibrils on the entire surface of the SCO and then into the RF (Sterba et al., 1967; Rodriguez et al., 1992). Then, RF continuously grows by the addition of new SCO-spondin molecules at the distal end, which disassembles at the caudal region of the spinal cord where it forms a flocculent material that enters into the local blood vessels (Rodriguez et al., 1992; Molina et al., 2001; Troutwine et al., 2020).

The role of RF in body axis architecture determination was first proposed more than a century ago by Nicholls (1917) after the observation that RF incision in fish generates its retraction and abnormal posture (Nicholls, 1917). This relationship was also observed in lordotic fish that present anomalies in the central canal, RF, and SCO secretory activity (Andrades et al., 1994). The use of zebrafish mutants has confirmed the crucial role of RF in body axis maintenance. By using CRISPR/Cas9-mediated genome editing, Cantaut-Belarif et al. (2018) generated a *scospondin* (*sspo*) mutant that cannot assemble the RF. Such a mutant embryo develops a curly tail down phenotype, which gradually worsens until death occurs 10 days after fertilization (Cantaut-Belarif et al., 2018). However, removing the chorion of the *sspo* null mutant early embryos allows the survival of 30% of them, which mature into adult fish with a severe curved body axis (Lu et al., 2020).

Although the *sspo* mutants resemble the curly tail down phenotype seen in cilia motility mutants, the cilia activity and CSF flow are normal in *sspo* mutants (Cantaut-Belarif et al., 2018). By contrast, the secretion of SCO-spondin is apparently normal in mutants with defective cilia, but the RF fails to develop (Troutwine et al., 2020). Altogether, this data suggests a crucial role of motile cilia in SCO-spondin aggregation and RF formation.

Since the *sspo* null mutation generates a fully penetrant curvy body axis phenotype, complementary studies have focused on the use of mutants showing incomplete penetrance (Table 1). In a zebrafish forward genetic screen for adult viable scoliosis mutants, Troutwine et al. (2020) studied two recessive hypomorphic alleles harboring missense mutations in *sspo*. In contrast to null *sspo* alleles, the RF of hypomorphic mutants appeared devoid of defects during the initial assembly phase, but it progressively became abnormal until its disappearance at 10 days post-fertilization (dpf), at the same time that appears the curved axis phenotype (Troutwine et al., 2020). In addition, the generation of a SCO-spondin-GFP fusion transgenic line has facilitated genetic and phenotyping efforts, allowing to monitor *in vivo* the dynamic properties of RF formation in developing zebrafish embryos (Troutwine et al., 2020). This transgenic line has revealed unprecedented features of the initial SCO-spondin secretion and RF formation at the rostral portions of the spinal canal. Taken together, the analysis of SCO-spondin zebrafish mutants, displaying curvy tail down phenotypes, shows that the RF disassembly coincides with the development of an axial curvature of the spine (Cantaut-Belarif et al., 2018; Troutwine et al., 2020). The relevance of RF in the adult has also been reported in *sspo* null mutants that manage to survive after dechorionization, and exhibited a strong curvature in the spine (Lu et al., 2020; Rose et al., 2020). Also, in heterozygous *sspo* ^dmh4/+^ mutants that, as well as AIS patients, develop the spinal curvature during juvenile stages (Rose et al., 2020). These findings support the relevance of RF to maintain right body axis, and open the question about the function exerted by this fiber in the central canal of the spinal cord.

**TABLE 1:** Molecular lesions in the zebrafish *scospondin* (*sspo*) gene.

Mutant allele	Mutation	References
*scospondin* ^*icm13*^	Frameshift mutation results in a truncated protein devoid of any domain	Cantaut-Belarif et al. (2018), Lu et al. (2020)
*scospondin* ^*icm15*^	Insertion of five extra amino acids at the EMI domain that prevents multimerization and aggregation of RF	Cantaut-Belarif et al. (2018), Orts-Del’Immagine et al. (2020)
*scospondin* ^*dmh4/+*^	Missense mutation results in a cysteine to phenylalanine substitution at the TIL domain, causing abnormal Sspo function	Rose et al. (2020)
*scospondin* ^*stl300*^	Hypomorphic mutation that disrupts a conserved cysteine residue leading to disassembly of the RF	Troutwine et al. (2020)
*scospondin* ^*stl297*^	Hypomorphic mutation that disrupts a conserved cysteine residue leading to disassembly of the RF	Troutwine et al. (2020)

#### 4.3.2 The Link Between Reissner Fiber and Cerebrospinal Fluid Contacting Neurons to Conform a Proprioceptive Organ in the Zebrafish Spinal Cord

As stated before, the neuropeptides Urp1 and Urp2 are expressed in CSF-cNs, which are localized in the zebrafish spinal cord (Zhang et al., 2018) (Figure 2A). Interestingly, these molecules are down-regulated in the *zmynd10* mutant and other cilia zebrafish mutants (Zhang et al., 2018) and their overexpression can rescue body axis curvature in different cilia mutants. These findings indicate that the cilia-driven CSF flow would be necessary to trigger Urp signaling in CSF-cNs and these molecules then induce body straightening in zebrafish embryos. Additionally, a transcriptomic analysis of *sspo* mutants revealed a high down-regulation of Urp2, and in a lesser extent of Urp1(Cantaut-Belarif et al., 2020; Rose et al., 2020); and the restoration of Urp expression in CSF-cNs of *sspo* mutants showed a significant rescue of embryonic body curvature (Lu et al., 2020). These antecedents show that the expression of Urps in CSF-cNs is sufficient to restore axial deformities in *sspo* mutant and in motile cilia mutants (Zhang et al., 2018; Lu et al., 2020).

CSF-cNs are sensitive ciliated cells surrounding the central canal of the vertebrate ventral spinal cord (Jalalvand et al., 2014; Djenoune et al., 2017). These GABAergic neurons detect local spinal bending and relay this mechanosensory feedback information to motor circuits involved in locomotion and postural control (Bohm et al., 2016). In this way, the genetic blockage of neurotransmission in CSF-cNs by expression of botulinum toxin light chain under the specific promoter of the Pkd2l1 channel, reduces locomotor frequency and hampers postural control (Hubbard et al., 2016). In zebrafish larvae, CSF-cNs send an ipsilateral ascending axon from two to six segments that synapse onto premotor interneurons involved in slow locomotion, onto primary motor neurons that innervate the entire ventral musculature, and interneurons involved in sensory-motor gating (Figure 2C). Moreover, the rostral most CSF-cNs contact large reticulospinal neurons of the hindbrain, that in turn send descending motor commands to the spinal cord (Figure 2C) (Wu et al., 2021), suggesting that the contractile activity of these structures promotes proper axial morphogenesis (Zhang et al., 2018). Additionally, adult *pkd2l1* zebrafish mutant develop a moderate spine curvature but did not resemble the motile cilia mutant phenotype. The opening of Pkd2l1 channel is regulated by mechanical pressure applied against the membranes of CSF-cNs (Cantaut-Belarif et al., 2018), that may reflect the CSF flow or even the RF contact. Changes in CSF pH and osmolarity can also affect the functionality of these neurons, impacting on the posture through spinal curvatures due to inadequate muscle contraction (Orts-Del’Immagine et al., 2014; Bohm et al., 2016; Jalalvand et al., 2016a; Jalalvand et al., 2016b). These antecedents reveal that these CSF-cNs contribute to the maintenance of natural curvature of the spine (Sternberg et al., 2018).

Morphologically, CSF-cNs project a ciliated apical extension in the central canal that may contact the RF and detect spinal curvature in a directionally motion-directed manner (Orts-Del’Immagine et al., 2020). Using a hypomorphic mutant of the *sspo* gene, Orts-Del’Immagine et al. (2020) revealed that in the absence of RF, CSF-cNs reduce their ability to allow calcium into these cells. In addition to mechanical stimuli, this neural population is also chemosensitive since it responds indirectly to CSF norepinephrine (Orts-Del’Immagine et al., 2020). Interestingly, it has been reported that RF binds and transports norepinephrine (Figure 2A) (Caprile et al., 2003), likely linking the mechanosensitive and chemosensitive responses of CSF-cNs. However, the role of Urp2 expression in these neurons is unclear. Rose et al. (2020) emphasized that this peptide is down-regulated in *sspo* mutants, but that its expression levels were not back to normal by inhibiting the pro-inflammatory factor COX-2 that rescues the mutant axial development (Rose et al., 2020). In addition, overexpression of Urp2 in CSF-cNs of *sspo* mutants rescued curvature phenotypes during the larval-to-adult transition, thus concluding that its expression is sufficient to restore body axis straightening of zebrafish individuals showing both ciliopathy and RF abnormalities (Lu et al., 2020). Additionally, the expression of Urp2 in *sspo* mutants can be restored by the exposition to adrenaline and noradrenaline (Cantaut-Belarif et al., 2020; Lu et al., 2020), neurotransmitters that are normally attached to the RF (Caprile et al., 2003).

Altogether, these findings indicate that, at least in zebrafish, the maintenance of the spinal right axis requires the cilia motility to promote a correct CSF flow, which in turn is necessary for the RF assembly to transport different signaling molecules (i.e., norepinephrine) along the entire nervous system (Figure 2A). Depending on the spinal position, RF would contact the cilia of the CSF-cNs (Figure 2B), which finally synapse onto motor neurons, premotor excitatory or reticulospinal neurons; thus, modulating the excitability of spinal circuits underlying locomotion and posture (Figure 2C). Ultimately, mutations of any gen that perturbs this cascade of events will lead to spinal curvature. Having this information, the question that arises is whether these phenomena also occur in humans, and their possible connection with AIS.

#### 4.3.3 Human Idiopathic Scoliosis and Its Link With Cerebrospinal Fluid Flow, Ciliopathies, and an Altered Proprioception

The transparency of the zebrafish larvae facilitates the visualization of CSF flow, RF dynamics and CSF-cNs connectivity, elements that conform a proprioceptive organ involved in locomotion and postural control and is also required for the maintenance of natural curvature of the spine (Figure 2). The presence of this spinal proprioceptive organ in other vertebrates has not yet been proved. However, the literature suggests that, except for humans, similar processes may be occurring in vertebrates, as they have the same anatomical elements. Thus, it has been described in most vertebrates the presence of a central canal in the spinal cord, surrounded by ciliary ependyma cells and CSF-cNs (Djenoune and Wyart, 2017), and containing the RF inside (Rodriguez et al., 1984).

On the other hand, there are arguments suggesting that in humans this proprioceptive organ would not be present, or it will have some differences. Firstly, the central canal of human spinal cord, in embryos and children, is similar to the one described in other vertebrates (Sakakibara et al., 2007). After this period, it becomes discontinuous and from the second decade of life it becomes obliterated and contains a morphologically heterogeneous accumulation of cells (Garcia-Ovejero et al., 2015). In this way, it is important to highlight that the biological material under study is obtained from autopsies, in which the spinal cord can only be dissected several hours or days after death. For this reason, the occlusion of the canal central has been largely interpreted as postmortem artifact. However, magnetic resonance imaging (MRI) studies of more than hundred individuals confirms that the central canal is absent from the vast majority of individuals beyond 18 years old, gender-independently and throughout the entire length of the spinal cord (Garcia-Ovejero et al., 2015). Secondly, although the principal component of the RF, SCO-spondin, has been described solubilized in human fetal CSF (Ortega et al., 2016), there are not reports showing the RF in the human spinal cord. In fact, the gland that secretes this protein, the SCO, suffers a progressive atrophy after childhood and is only vestigial in adult individuals (Rodriguez et al., 2001). Finally, the presence of CSF-cNs has been described in more than 200 vertebrates (reviewed in Djenoune and Wyart, 2017), but to our knowledge there are no reports showing these cells in the human spinal cord.

In spite of these differences between humans and other vertebrate organisms, there are also antecedents suggesting that cilia and spinal CSF flow are important to maintain the spinal curvature and that disorders in the proprioception are a cause of AIS. A recent study using a noninvasive and radiation-free MRI revealed disturbances in the CSF flow into the spinal cord in scoliotic individuals, especially at the thoracic level (Algin et al., 2021). This finding is promissory, although these authors analyzed the velocity of the subarachnoid CSF. Additionally, the authors mention that these alterations in the CSF flow in patients with scoliosis may be related to changes in the thoracic cavity or respiratory disturbances. It will be interesting to increase the number of individuals analyzed to know if subarachnoid CSF disturbances described into the spinal cord is the cause or a secondary effect of scoliosis.

A compromised CSF flow in human scoliosis has also been suggested in individuals with Chiari malformation type I (CM-I) with or without syringomyelia (Pinna et al., 2000; Panigrahi et al., 2004). Indeed, scoliosis is the most common deformity found in these patients, with an incidence of up to 20% (Colombo and Motta, 2011; Kelly et al., 2015). CM-I is characterized by descent protrusion of the cerebellar tonsils into the cervical spinal canal, generating a partial obliteration and impeding the free CSF movement at the cranio-cervical region (Buell et al., 2015). The prevalence of scoliosis in these patients depends on the degree of CSF flow obstruction, independent of the size of the cerebellar tonsil herniation (Shaffer et al., 2014). The current treatment proposed for CM-I patients consists in correcting the CSF flow by decompression of the cranio-cervical region, leading to the reestablishment of the normal body axis in young patients (Kelly et al., 2015). It is important to highlight that the obstruction of the CSF flow in CM-I individuals has also been studied at the subarachnoid level instead of at the central canal as performed in zebrafish studies.

In relation with the link between cilia and scoliosis in humans, is important to separate between motile cilia and primary cilia disturbances. In this way, PCD is characterized by an alteration in motile cilia, leading to chronic respiratory tract infections, abnormally positioned internal organs, and infertility (reviewed in Mirra et al., 2017). However, this pathology is not clearly associated with body axis maintenance. WES studies from two women of consanguineous families with PCD revealed that the mutation of the dynein axonemal assembly factor 2 (DNAAF2) gene may also be associated with spine malformation (Lu et al., 2021). Both patients exhibited bronchiectasis, sinusitis and infertility, classical disorders in PCD, and one of them also presented situs inversus and scoliosis. However, the spinal alteration found in this patient seems to be a consequence of the situs inversus (Schlosser et al., 2017), and not a direct consequence of the cilia motility or alterations in CSF flow.

Regarding to primary cilia function, the first WES analysis performed in diverse families affected with IS identified three different variants of the POC5 gene, which co-segregated with the disease (Patten et al., 2015). To analyze the function of this gene, a mutant version of the *poc5* transcript was injected into zebrafish one- or two-cell stage, generating an overt axial phenotype after 72 hpf, ranging from mild to severe curvature of the body axis in approximately 50% of the injected embryos (Patten et al., 2015). *In situ* hybridization analysis performed in these embryos revealed that at 72 hpf stage, when the curved phenotype appears, this gene is expressed mainly in the brain but not in the spinal cord. The authors suggested that this mutation may be impairing the function of primary cilia, and it was confirmed in a posterior analysis in which cells that express the mutated *poc5* gene have shorter cilia (Hassan et al., 2019).

In a similar report, WES analysis from patients with IS with a familiar history revealed a heterozygous variant within the ciliary kinesin family member 7 (KIF7) gene (Terhune et al., 2021a). This gene was studied in control and other IS patients, showing a different mutation in affected individuals with IS only. This finding leaded to the generation of a *kif7* zebrafish *crispant*, which displayed severe scoliosis, present in juvenile individuals and progressing through adulthood. It is important to mention that these animals had an intact CSF flow and RF structure (Terhune et al., 2021a). By contrast, the expression of some genes related to the hedgehog (Hh) signaling is altered in *kif7* mutants, suggesting a connection between the *kif7* gene function and Hh pathway. Another gene, also linked to AIS susceptibility and primary cilia function, is the tubulin tyrosine ligase like gene member 11 (TTLL11) (Mathieu et al., 2021). This protein induces tubulin glutamylation, required for cilia elongation. The function of this gene was studied in a zebrafish model, and the resulting phenotype showed spinal deformity at larvae and adult stages (Mathieu et al., 2021).

In summary, sequence alterations of these three genes (POC5, KIF7 and TTLL11) found by WES analysis from scoliotic individuals suggest disturbances of primary cilia function. The posterior generation of zebrafish mutants confirmed the relevance of these genes in the maintenance of a straight body axis. In this way, the massification of WES analysis and the ease performing of CRISPR/Cas9-mediated targeted mutagenesis in zebrafish, are providing us with a powerful tool to understand the pathophysiology of human diseases, particularly scoliosis.

Other mutations affecting primary cilia function (revised in Oliazadeh et al., 2017) have been described testing the association between rare variants and scoliotic phenotypes in GWAS studies. As mentioned before, the primary cilia is present in many cell types, sensing their environment, and capable of responding to fluid flow dynamics. For example, primary cilia participate in mechanotransduction and are a crucial part of the vestibular region in the inner ear (Whitfield, 2020), a proprioceptive organ capable of detecting gravity, acceleration, and orientation of the body (similar to the proprioceptive organ formed by CSF-cNs and RF in zebrafish). This detection requires the tethering of calcified masses to the cilia of mechanosensory hair cells. The mammalian ear contains thousands of biomineralized particles called otoconias, whereas the inner ear of zebrafish contains three large ear stones called otoliths that execute a similar function (Petko et al., 2008). A defect in the otolith formation is characteristic in most of the zebrafish ciliary mutants and morphants (Whitfield, 2020).

The link between AIS and vestibular disturbances has been well documented (Wiener-Vacher and Mazda, 1998; Pollak et al., 2013; Hitier et al., 2015; Antoniadou et al., 2018; Hatzilazaridis et al., 2019), suggesting that in response to the asymmetry between the left and right vestibular organs, the individuals adopt an asymmetric posture and activation of the axial muscles that in turn generates a spine deformity. This bilateral organ is highly conserved in vertebrates and projects to various spinal cord level, forming the lateral and medial vestibule-spinal tracts, the tangential vestibular nucleus and vestibule-reticule-spinal projections, controlling motor targets at different spinal levels (reviewed in Deliagina et al., 2014; Gordy and Straka, 2021). The relevance of this proprioceptive organ in the maintenance of the body axis has been demonstrated in different vertebrates, such as *Xenopus*, in which the unilateral removal of this organ generates a severe curvature in the spinal cord (Lambert et al., 2009). In this way, the zebrafish has been proposed as a suitable model to study the vestibular function (Bagnall and Schoppik, 2018). Finally, it is interesting to note that the expression of Otoc1, a protein required for the correct otolith formation, coincides almost exactly with the expression pattern of SCO-spondin (Petko et al., 2008). Also, Otogelin, a protein required for otoliths tethering to the hair cells has identical SCO-spondin’s functional domains, as revealed by comparing their amino acid sequences (VS and TC, unpublished).

## 5 Mechanisms of Inflammation and Scoliosis: Emerging Models of an Immune Response-Mediated Spine Curvature Regulation

Inflammation is a physiological process responsible for restoring structural and functional homeostasis in tissues altered by different harmful stimuli, including infectious, mechanical, chemical, or physical factors (Serhan et al., 2007; Takeuchi and Akira, 2010; Klein-Wieringa et al., 2016). Also, inflammation is characterized by classic symptoms such as heat, pain, swelling and redness, among others (Klein-Wieringa et al., 2016). Although AIS causes remain controversial, studies into the molecular mechanisms of spine formation have revealed an active involvement of the inflammatory response in the development of this highly prevalent structural disorder (Table 2). However, while the influence of inflammation as a trigger of AIS development remains unclear, some studies indicate that infection and altered immune mechanisms might be correlated with this pathology (Papanastasiou et al., 2010; Sanders et al., 2012; Van Gennip et al., 2018), as well as the appearance of scoliotic phenotypes (Klein-Wieringa et al., 2016). In the next section, we will discuss how infectious agents, external stressors, and inflammatory responses promote scoliosis and spine malformations in zebrafish.

**TABLE 2:** Inflammatory responses and their implications in AIS pathogenesis.

Impaired immune response	Target tissue damage	Altered mechanism	Triggering effect of IS	References
Fluid dynamics alteration	Cerebrospinal fluid	• Chemical and mechanical signaling of the fluid• Immune cell infiltration	◦ Failure in muscle contraction ◦ Altered myelination process ◦ CSF-cNs communication damaged	Acaroglu et al. (2009), Hayes et al. (2014), Hubbard et al. (2016), Van Gennip et al. (2018)
Chronic inflammation	Muscles Nerves Bones	• STAT3 damagedfluid• Change from inflammatory to anti-inflammatory responsefluid• Lymphocyte differentiation	◦ Increased osteoclast activity ◦ Damaged collagen genes ◦ Nerve damaged	Minegishi et al. (2007), Papanostasiou et al. (2010), Sanders et al. (2012), Xiong et al. (2017)
Excessive inflammatory response	Extracellular membrane	• Cell adhesion failuresfluid• Failure of intracellular signaling mechanisms	◦ Morphological and functional changes in tissue ◦ Cellular apoptosis ◦ Increase in mediators (cytokines, EROs, PGE2, MMPs, among others)	Haller et al. (2016), Karner et al. (2015), Tenor et al. (2011)
Increased phophodiesterase 4 activity	Cartilage	• Increase in nitric oxide (NO) production	◦ Cartilage tissue destruction	Tenor et al. (2002)
Increased microglial activity	Nerves	• Schwann cells damage	◦ Neuromuscular communication ◦ Failure in myelination process	Monk et al. (2011), Mogha et al. (2013), Van Gennip et al. (2018), Desplats et al. (2020)

### 5.1 Infectious Agents and Spine Malformations

It has been shown that infections by pathogens, such as the fungus *Pseudoloma neurophilia*, affect the nervous and muscular system, generating scoliosis in zebrafish (Sanders et al., 2012). The infection produced by *P. neurophilia* is chronic and difficult to control. Indeed, when the inflammatory process is perpetuated, the action of immune cells to eliminate this fungus through phagocytosis releases oxidative substances and hydrolytic enzymes, which can harm the zebrafish body (Nathan, 2002; Camicia and de Larranaga, 2013; Serhan, 2014). Ultimately, generates an abnormal phenotype consisting of evident signs of spinal deformities and slimming (Matthews et al., 2001).

On the other hand, the Hyper-Immunoglobulin E immune disease, which is characterized by excessive production of E antibodies and recurrent infections by the *Staphylococcus aureus*, has non-immune symptoms associated with the development of scoliosis (Grimbacher et al., 1999). However, recent pathophysiological studies of this disease have shown an alteration in the binding capacity of the signal transducer and activator of transcription 3 (STAT3) to target DNA sequences, thus limiting the response to the stimulation of interleukin 6 (Papanastasiou et al., 2010). STAT3 regulates the passage of the inflammatory to an anti-inflammatory response (Levy and Lee, 2002; Hillmer et al., 2016; Sorg et al., 2020) and zebrafish mutants show a dysfunction expression of collagen genes during the pathogenesis of scoliosis (Xiong et al., 2017). This highlights the importance of STAT3 as a regulator of various responses, including inflammation, for the correct development of the vertebral axis.

### 5.2 Inflammation, Oxidative Stressors, and Neurodegenerative Processes Involved in Zebrafish Scoliosis

In addition to pathogens, oxidative stressors are involved in scoliosis generation. For instance, Tenor et al. (2002) showed that up-regulation of phosphodiesterase 4 (PDE4) in chondrocytes influenced by an inflammatory environment led to increased nitric oxide (NO) levels. The degradation of cartilage, and a subsequent spine malformation, was accelerated in response to interleukin-1β *in vitro* through known mediators, including prostaglandin E2, NO, and metalloproteinases (Tenor et al., 2002; Tenor et al., 2011). In zebrafish, recent studies have found that *ptk*7 mutants also exhibit up-regulation of reactive oxygen species-related genes, suggesting a link between oxidative stressors and scoliosis. Interestingly, when juvenile zebrafish *ptk7* mutants were treated with the antioxidant agent N-acetylcysteine (NAC), the incidence of scoliosis and the severity of spinal curvature were considerably reduced (Van Gennip et al., 2018). This confirms the interplay between oxidative stressors and the development, as well as the severity, of spine malformations. In addition, the study of zebrafish *sspo* mutant larvae has provided significant progress in our understanding of the link between oxidative stressors and scoliosis. In *sspo* mutants, the high mortality due to the development of a curly tail down phenotype is largely rescued by NAC ethyl ester (NACET) treatment, suggesting that an oxidative stress-dependent immune response controls the degree of scoliosis in zebrafish (Rose et al., 2020). Remarkably, the use of antioxidants and anti-inflammatory agents reverse the morphological alterations of the body axis. Therefore, we foresee that future efforts will be crucial to better understand the effect of oxidizing agents in scoliosis development.

Several studies have also indicated that neurodegenerative demyelination processes occur with episodes of stress and chronic inflammation (Carter et al., 1995; Feehan and Gilroy, 2019; Desplats et al., 2020). For instance, failures in myelination increase microglial activation, inflammation, and spine malformation in vertebrates (Monk et al., 2011; Mogha et al., 2013). Low levels of Leptin, an enzyme involved in myelination, have been linked to a high risk of scoliosis in adolescents (Qiu et al., 2007; Liang et al., 2012; Liu et al., 2012). Additionally, Leptin participates in the differentiation and mineralization of the bone matrix, promoting the proliferation of osteoblasts and chondrocytes, and inhibiting osteoclastic activity (Wu et al., 2012; Man et al., 2019). Increases in osteoclast differentiation and functionality have been observed in cases of scoliosis, also associated with the incorrect activation of STAT3 (Minegishi et al., 2007).

There is a growing interest in studying the effect of oxidizing agents in scoliosis development. This, can be conducted by using genetic and pharmacological approaches. Thus, the identification of oxidative and neurodegenerative stress markers in zebrafish models of AIS emerges as a valuable tool to investigate how stress-responsive mechanisms are involved in spine morphogenesis.

### 5.3 Cerebrospinal Fluid and Inflammatory Response Involved in Zebrafish Scoliosis

**FIGURE 3:**
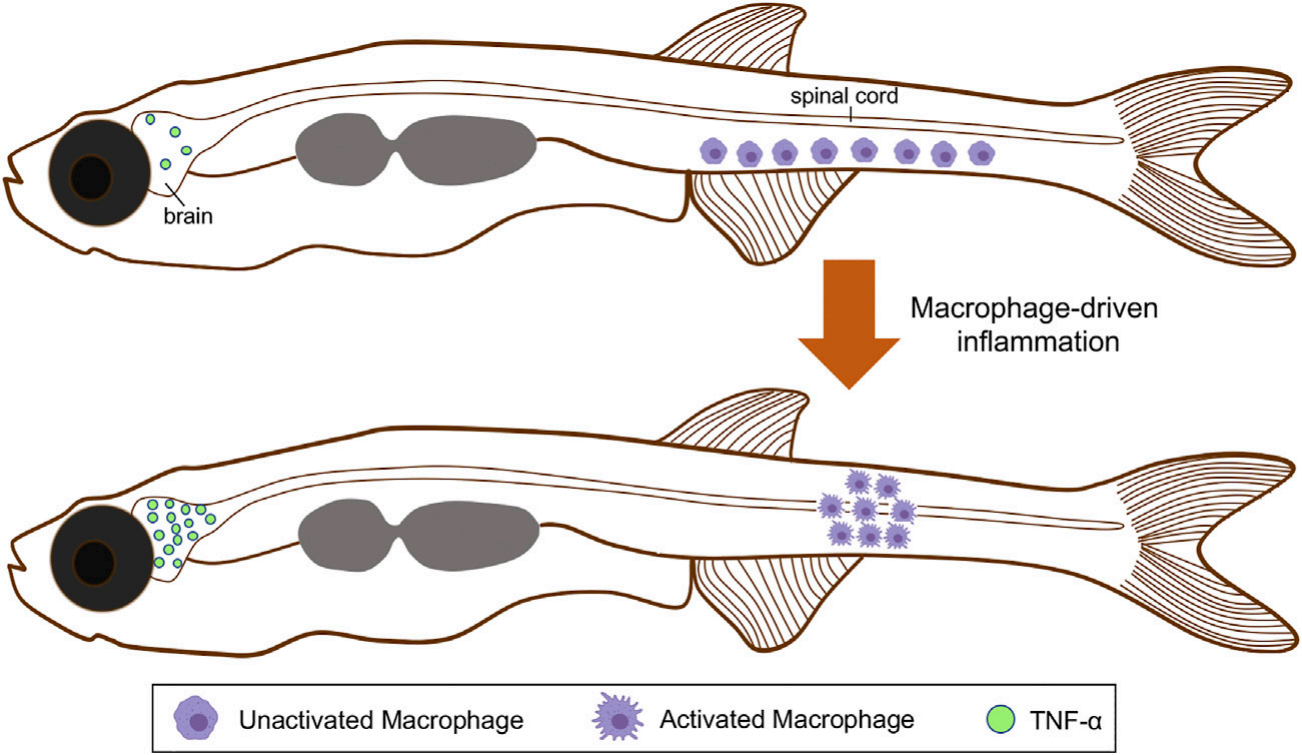
Schematic representation of macrophages activation and spatial distribution, and the regionalized inflammatory response triggered during scoliosis development. Colored macrophages (purple) are shown. At the onset of the spinal curve formation, immature macrophages are distributed along the ventral side of the tail. After their activation, these cells move and accumulate within the spinal cord. Macrophages maturation also triggers a neuroinflammatory response associated with TNF-α overexpression in the brain. TNF-α, tumor necrosis factor alpha.

As discussed earlier, the etiology of AIS has been associated with functional disturbances in genes related to cilia motility, CSF flow, and inflammatory response (Van Gennip et al., 2018). Over-expression of proinflammatory factors, such as tumor necrosis factor alpha (TNF-α), confirmed a neuroinflammatory response in *sspo* zebrafish mutants, both at embryonic (3 dpf showing a curvy tail down phenotype) and post-embryonic stages (Figure 3) (Rose et al., 2020). *In vivo* monitoring of immune cells showed that the accumulation of macrophages in the curved areas, specifically within the medullary zone (Figure 3). Conversely, the migration of macrophages and neutrophils to the telencephalon was visualized in the zebrafish *sspo* mutant larvae in association with high expression of the *tnf-α* gene (Rose et al., 2020). In addition, transcriptomic and differential gene expression analysis of brains isolated from 21 dpf heterozygous *sspo* mutants revealed that inflammation-related genes were up-regulated compared to wild-type siblings (Rose et al., 2020). Similar results were found in the *ptk7* mutant (Van Gennip et al., 2018).

Furthermore, the relevance of the inflammation response triggered by a spinal curvature was demonstrated through anti-inflammatory drug treatment, which suppressed the severity and incidence of spine defects in cilia *ptk7* mutants (Van Gennip et al., 2018). In the same way, the treatment of zebrafish *sspo* mutant embryos with a cyclooxygenase-2 (COX-2) inhibitor can fully rescue normal axial development, even though the same animals developed scoliosis during early juvenile stages (Rose et al., 2020).

Also, the zebrafish *ptk7* mutant displaying impaired CSF movement and ciliary structure also exhibits inflammatory and demyelinating events (Hayes et al., 2014). These data support the idea that an inflammatory response is activated in the development of scoliosis caused by the alteration of CSF flow (Van Gennip et al., 2018; Rose et al., 2020). On the other hand, the arrival of cellular reinforcements and other mediators during inflammation can alter the properties of the CSF. Thus, changes in the osmolarity between CSF interstitial and blood fluids occur depending on the proximity to the inflammatory focus, negatively affecting communication signals necessary for the correct posture of the vertebral axis (Klarica et al., 2013; Bohm et al., 2016).

Altogether, these studies indicate that inflammation play an important role in disturbing the homeostasis of various signaling pathways in generating spinal curvatures. Therefore, it is necessary to pursue the study of inflammation pathways during the development of scoliosis. Such an approach will undoubtedly identify novel factors controlling spinal morphology, coupled with functional monogenic interrogation in vertebrate animals (e.g., zebrafish) will provide new ways of preventing and treatment for AIS in humans.

## 6 Phenogenetic Data Integration and Scoliosis Modeling in the Post-Genomic Era

Currently, the availability of complete genome sequences of many model organisms is allowing us to evaluate gene function and decipher the complex crosstalk between genomics and phenomics. In this quest, deep phenotyping is a valuable approach to capture subtle and dynamic cellular and molecular variations (Bilder et al., 2009; Brown et al., 2018; Fuentes et al., 2018). This consists of a multi-scale experimental approach aiming at searching, measuring, and analyzing the biological origins of quantifiable traits; thus, unraveling genotype-phenotype associations and explaining how genomic information is expressed to determine complex morphological features. In this sense, zebrafish is amenable for gene function discovery, and efficient and rapid phenotyping; therefore, it represents an ideal multicellular model for deep phenotyping (Fuentes et al., 2018).

Traditional and automated histological techniques such as high-throughput histology and micron-scale computed tomography (microCT) have recently been used to describe mutant phenotypes, including those showing alterations in skeleton morphogenesis. Thus, whole-mount staining and high-resolution imaging of juvenile and adult animals are being exploited to generate precise phenogenetic information of organ systems and tissue morphology as well as physiological defects at cell resolution level, including the spine architecture (Sabaliauskas et al., 2006; Charles et al., 2017; Henke et al., 2017; Hur et al., 2017; Weinhardt et al., 2018; Ding et al., 2019; Kwon et al., 2019; Marie-Hardy et al., 2019). This, has been pivotal to understand gene function and AIS etiology caused by single gene lesions (Grimes et al., 2016; Gistelinck et al., 2018; Zhang et al., 2018; Gray et al., 2020; Lu et al., 2020; Rose et al., 2020; Sun et al., 2020; Troutwine et al., 2020).

Although microCT imaging has allowed quantitative morphometric analysis of the spine defects in zebrafish AIS-like models, it has also revealed subtle phenotypic deviations that would not be observable by the naked eye. The study of post-embryonic features by using deep skeletal phenotyping identified spatial bone mineralization patterns within the axial skeleton (Hur et al., 2017). For instance, computational-based morphological and densitometric analysis of mutant phenotypes demonstrated the value of linking interrogation of genes requirements and phenomic profiling (Hur et al., 2017).

In addition to mutagenesis approaches, complementary high-resolution and -quality 3D axial skeleton reconstructions will help determine the morphological effects of manifested mutations generating spine malformations (Hur et al., 2017; Weinhardt et al., 2018; Ding et al., 2019; Kwon et al., 2019; Marie-Hardy et al., 2019). Importantly, sequencing technologies will reveal a list of zebrafish genes and genetic pathways for phenomic analysis to systematically describe and define their function. In this context, the zebrafish model is advantageous because: i) it is genetically tractable and there is a broad range of manipulation methods, most of them relatively easy to perform, and ii) it is possible to conduct molecular manipulations that can be followed over time through geometric morphometrics approaches. Thus, patterns of deformations can be detected and deliver data to model spine biomechanical performance in the context of a living vertebrate organism for being extrapolated to human AIS studies.

## 7 Discussion

Scoliosis is the most common deformity of the spine in humans. Among the different types of scoliosis, AIS represents a predominant spine deformity worldwide. Since the first report of AIS in humans (Wynne-Davies, 1968), our knowledge of such spine malformation has increased considerably, especially in the cellular, molecular, and genetic aspects. On the other hand, with the dawn of the genomic revolution, there has been a substantial increase in our knowledge of the molecular nature of genes and their function associated with scoliosis. Despite the use of a wide diversity of animal model systems used to study AIS, the etiology of this spine deformity is far away to be understood. This, has been partially caused by the lack of animal models that recapitulate the genotype-phenotype relationships to unravel the etiology of AIS. With the aim to identify current knowledge gaps around AIS and propose future animal model systems to investigate this condition, we here focused on what is known about the cellular, molecular, and functional processes underlying spine malformations in zebrafish. We highlighted the potential of zebrafish as a phenogenetic platform to understand the functional and molecular program underlying AIS-like phenotypes.

Several mechanisms and features of zebrafish spine deformity, such as the role of ciliogenesis, CSF flow, and inflammation, have been investigated in mutants to particular human AIS-related genes. Although the contribution of zebrafish as a genetic system has been crucial to study spine malformation, it is unclear if this animal model recapitulates the pathogenesis cause for all known human AIS risk loci. Further knowledge of the genetic factors controlling the spine formation is critical to understand the etiology of AIS. By doing so, it will reveal candidate genes associated to this malformation and allow the development of new therapeutic alternatives to improve the quality of life of humans worldwide.

The progress in the study and definition of genetic risk factors for human scoliosis appears to be limited in the ability to link specific genomic sequences with the disease, which could increase clinical biases and imprecise genotype-phenotype associations. High throughput next-generation sequencing has unveiled the dysregulation of thousands of non-coding RNAs, including microRNAs (miRNAs) and long non-coding RNAs (lncRNAs), linked to scoliosis (Garcia-Gimenez et al., 2018; Li et al., 2020). Indeed, recent studies using blood samples of patients with AIS have detected dysregulated miRNAs levels in humans (Ogura et al., 2017; Garcia-Gimenez et al., 2018). Further analysis of their targets and activation of cellular pathways have indicated that miRNA imbalance could change the homeostasis of bone formation and destruction, and by doing so, ultimately contributing to the AIS pathology (Garcia-Gimenez et al., 2018). Another type of non-coding RNAs with a putative role in scoliosis corresponds to lncRNAs. A comprehensive screening of lncRNA and mRNAs in AIS patients showed that 139 lncRNAs were differentially expressed compared with healthy controls (Liu et al., 2015). The aforementioned link between ncRNAs and scoliosis has revealed new information from AIS patients. Thus, a great number of these dysregulated molecules could represent an additional layer of regulation and potential diagnostic biomarkers of this multifactorial pathology. However, genetically tractable models to understand molecular genetics and identify conserved ncRNAs linked to AIS in humans are needed. Therefore, future phenotyping efforts are urgently needed to explore the role of these non-coding RNAs during spine zebrafish morphogenesis, which in turn will help determine the importance of these molecules in AIS.

In addition, the integration of disciplines, such as genomics and phenomics, into the study of zebrafish spine malformations will provide a description of the molecular, morphological, physiological and behavioral underpinning scoliosis/AIS. This will allow us to construct molecular genotype-phenotype maps that would be valuable resources to investigate not only this condition, but also other human diseases. In light of this, further efforts to construct molecular genotype-phenotype maps in zebrafish will provide guiding principles into human scoliosis/AIS. Taken together, we foresee that by using zebrafish, along with phenogenetic approaches would then in turn allow to progressively conduct novel therapies to treat scoliosis in humans.
